# Pituitary Teratoma: A Case Series of Three Cases

**DOI:** 10.7759/cureus.38729

**Published:** 2023-05-08

**Authors:** Martha L Tena Suck, Alma Ortiz Plata, Sergio Moreno Jimenez, Luis A Tirado García

**Affiliations:** 1 Neuropathology, Instituto Nacional de Neurología y Neurocirugía, Mexico City, MEX; 2 Neurosurgery, Instituto Nacional de Neurología y Neurocirugía, Mexico City, MEX; 3 Pathology, Instituto Nacional de Neurología y Neurocirugía, Mexico City, MEX

**Keywords:** sellar teratoma, skull base tumors, pituitary tumor, immunohistochemistry, mature teratoma

## Abstract

Mature cystic teratoma (MCT) is a benign germ cell tumor, histologically comprising components derived from mesoderm, ectoderm, and endoderm layer tissue. MCT usually has foci of intestinal components and colonic epithelia. Pituitary teratomas containing complete colon features are very rare. Here, we present three cases of sellar teratoma in two men aged 50 and 65 years and a woman aged 30 years. All patients presented with asthenia, adynamia, and loss of strength. A pituitary mass was incidentally observed on magnetic resonance imaging. Histological features showed a mature teratoma formed by gut and colonic epithelium, extended lymphoid tissue with the formation of Peyer’s patches, and muscular layer vestiges with a fibrous capsule. The immunohistochemical panel showed reactivity to cytokeratin (CK)7, CKAE6/AE7, carcinoembryonic antigen, octamer-binding transcription factor 4, cluster of differentiation (CD)20, CD3, vimentin, muscle actin, and *pituitary tumor-transforming gene 1* in isolated cells. However, alpha-fetoprotein, beta-human chorionic gonadotropin, human placental lactogen, CK20, tumor suppressor protein 53, and Kirsten rat sarcoma were negative. This article describes the clinical and histological features of rare sellar masses as well as survival after therapy.

## Introduction

Pituitary adenomas are among the most common central nervous system (CNS) tumors, representing an estimated 10-15% of all CNS tumors, and are the cause of approximately 25% of all surgical resections for these tumors [1]. Intracranial teratomas are rare and comprise about 0.5% of all intracranial tumors. They have a peak incidence during the first two decades of life and occur mostly in children [1], whereas germinomas occur in the early pubertal years. The neuroradiological findings indicate the presence of a mass with varying densities, typically containing both cystic and solid components, with inclusions such as teeth, fat, and calcification being suggestive findings [1]. Tumor markers such as beta-human chorionic gonadotropin (β-hCG) and alpha-fetoprotein (AFP) can be useful in diagnosing teratoma and distinguishing immature teratomas, mixed giant cell tumors (GCTs), and mature teratomas from immature or malignant components [1]. Recently, the new World Health Organization (WHO) classification categorizes them into neuroendocrine tumors, which include adenomas, and non-endocrine tumors, which encompass all other tumors [2]. Primary intracranial GCTs rather than teratomas are rare tumors, accounting for 0.5% of CNS tumors [3]. The most common intracranial location of GCTs is the pineal gland and suprasellar region [3,4]. Mature teratoma is a type of GCT, and the sellar-suprasellar mature teratoma has been rarely reported in the literature [4].

Here, we report three rare cases of mature cyst teratoma (MCT) that clinically mimicked a pituitary non-functional adenoma and histologically revealed colonic and gut features.

## Case presentation

Case one

**Figure 1:**
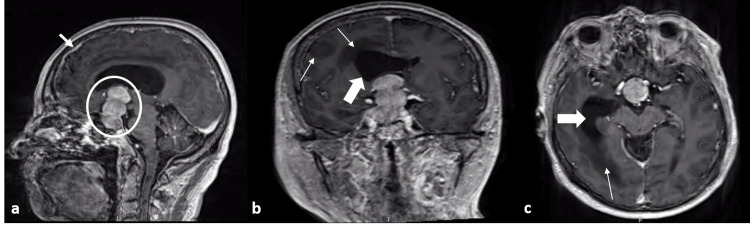
Imaging findings of case one. Magnetic resonance imaging reconstruction in T1 shows meningeal hyperintensity (arrow in a) and multiple hypointensities (thin arrows in b) in the parenchyma predominant in the right lobe. In the sella turcica, a homogeneous hyperintense mass with three well-rounded lobes can be observed (circle in a). It follows the medial line to the subventricular space causing ventriculomegaly (thick arrows in b and c).

A 65-year-old man presented to the institute with a two-month history of fatigue, nausea, vomiting, headache, drowsiness, limb weakness that caused an inability to walk, general loss of strength, physical deterioration, visual disturbances, and progressively developing amaurosis. He had suffered from alcoholism since the age of 34. Serum levels of AFP and carcinoembryonic antigen (CEA) were normal. Magnetic resonance imaging (MRI) showed meningeal hyperintensity as well as a homogeneous hyperintense mass in the sellar region with three well-rounded lobes that followed the medial line confined to the subventricular space causing ventriculomegaly. It was diagnosed as a non-functional pituitary macroadenoma. A transsphenoidal procedure was performed with adjuvant radiotherapy. He was discharged with no complications, and no follow-up was done. Imaging findings are illustrated in Figure 1.

Case two

**Figure 2:**
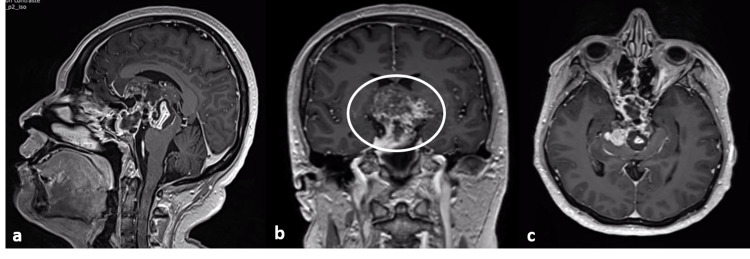
Imaging findings of case two. MRI reconstruction in T1 with gadolinium shows a diffuse heterogeneous sellar mass (a) with multiple densities and walls that give it a cyst-like appearance (oval in b). It pushes the adjacent structures and has a hyperintense capsule-like structure surrounding it (c).

A 50-year-old man presented to the institute with a four-month history of the same clinical presentation as case one. Serum levels of thyroid hormone were low with normal levels of AFP and CEA. The background was not relevant. MRI in T1 with gadolinium showed a diffuse heterogeneous sellar mass with a cyst-like appearance and a hyperintense capsule-like structure surrounding it. He was also diagnosed with a non-functional pituitary macroadenoma. A transsphenoidal procedure was performed, with no other therapy. He expired from neurological complications six months later. Imaging findings are illustrated in Figure 2.

Case three

**Figure 3:**
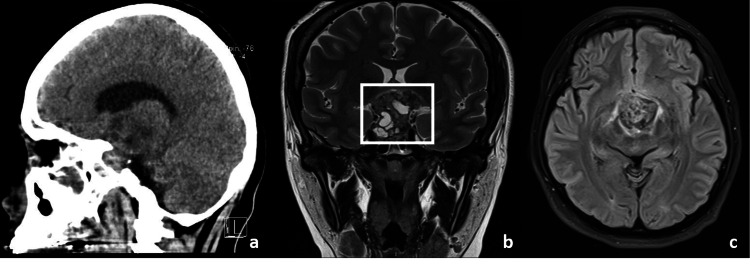
Imaging findings of case three. Sagittal computed tomography, coronal magnetic resonance imaging in T2, and a transversal fluid-attenuated inversion recovery (a, b, and c respectively) images show a sellar mass with multiple hypointense and hypodense areas, as well as with different densities in its content with walls that limit hyperintense content within a solid content (square).

A 34-year-old woman presented to the institute who six months after delivery complained of an inability to breastfeed, visual disturbances, headache, amenorrhea, drowsiness, depression, progressive loss of strength, and limb weakness that caused difficulty walking and standing. Serum prolactin levels were elevated, with normal AFP and CEA levels. MRI and computed tomography (CT) showed a sellar mass with multiple hypointense and hypodense areas, respectively, with different densities in its content and walls that limited a hyperintense content within a solid component. She was surgically intervened. Unfortunately, brain surgery was complicated by pneumonia and she succumbed 10 days later. Imaging findings are illustrated in Figure 3.

We present three cases of sellar teratomas in two men and one woman. All presented with headache, muscle weakness, and loss of tone and strength within a short duration (two to six months). The three patients had large tumors and showed heterogeneous imaging patterns, with one being cystic. Only case one received radiotherapy. No autopsy was performed. Results are presented in Table 1.

**Table 1:** Clinical features of the patients.

Clinical data	Case one	Case two	Case three
Demographics	65-year-old male	50-year-old male	34-year-old female
Symptom onset to presentation	Two months	Four months	Six months
History of teratoma	None	None	None
Clinical symptoms	Fatigue, nausea, vomiting, headache, drowsiness, difficulty walking, general loss of strength, and physical deterioration	Fatigue, nausea, vomiting, headache, drowsiness, difficulty walking, general loss of strength, and physical deterioration	Inability to breastfeed, headache, amenorrhea, drowsiness, depression, progressive loss of strength, and difficulty walking and standing
Visual disturbance	Progressive to amaurosis	Progressive to amaurosis	Yes postpartum
Alcoholism	31-year long	None	None
Hormone levels	Normal	Hypothyroidism	High prolactin
Diabetes insipidus	None	Yes	Yes
Serum alpha fetoprotein	None detected	None detected	Normal
Magnetic resonance imaging	Heterogenous mass (Hardy IIIE)	Heterogenous mass, cystic, with enhanced and fibrous cyst wall (Hardy IIIE)	Macroadenoma (Hardy IIIC)
Surgical procedure	Transnasal	Transnasal	Cerebral
Complication	None	Neurological disturbance. Deceased six months later	Pneumonia. Deceased 10 days later
Complementary treatment	Radiotherapy	None	None

**Figure 4:**
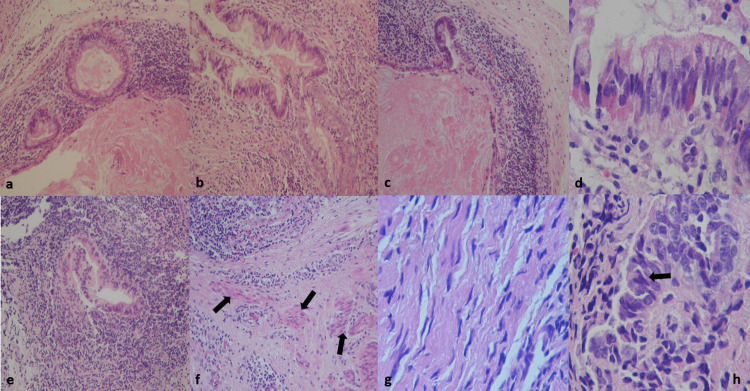
Histopathological features. The tumor was composed of isles of mucinous epithelium (a and b) (H&E ×200), cyst structures with goblet cells (c), forming true colonic and intestinal glands (d) with small cells that suggested lymphocytes (Peyer’s patches), which formed true lymphoid nodules (e). Muscular layer vestiges were also observed (arows in f), along with sheet nerve with undulate features (g) and small immature neuroepithelium (arrows in h) (H&E ×400). H&E: hematoxylin and eosin

Histological features of all cases showed a tumor formed by ectodermal, mesodermal, and endodermal components, with predominant colonic epithelial forming cyst structures (Figure 4a). Moreover, a few colonic glands were observed (Figure 4b). It was immersed in a dense infiltration of mature lymphocytes (Figure 4c), along with scaly epithelium with rare corneal bead formation (Figure 4d) and mucosal layer (Figure 4e), as well as a small focus of immature neuroepithelial (Figures 4f-4h) in case three with a thick fibrous capsule. These features are described in Table 2. Adeno or neurohypophyseal cells were not observed.

**Table 2:** Histological features.

Feature	Case one	Case two	Case three
Normal hypophysis	No	No	No
Adenoma	No	No	No
Fibrosis	++	+++	+++
Lymphocytes	++	++	+
Peyer’s patches	++	++	+
Bone tissue	++	+	No
Colonic gland	++	++	++
Skin epithelium	+	+	+
Sebaceous glands	+	+	No
Adipose tissue	+	+	+
Mature cartilage	++	+	+
Neuroepithelium	No	No	Yes
Mature cerebral tissue	No	No	No

**Figure 5:**
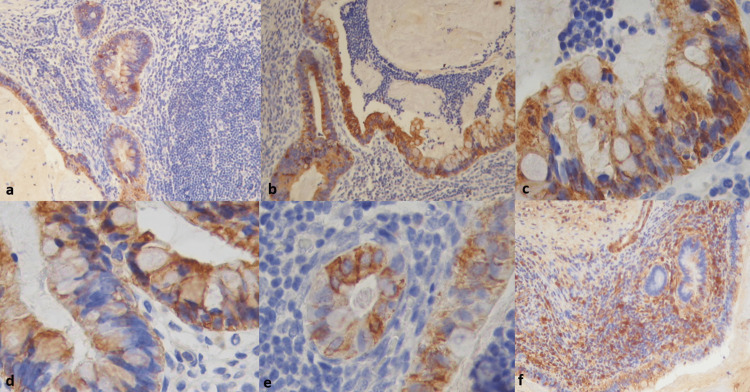
Immunohistochemistry features. Positive immunoreaction for CK7 in the mucous epithelium (a); positive immunoreaction for CKAE6/AE7 (b); positive for CK20 (c); CEA was also positive in the mucous epithelium (d); focal immunoexpression of PTTG-1 (e); CD20 was positive in some cells in the small cell layer formed by lymphocytes and Peyer’s patches vestiges (f) (original magnification ×400). CK: cytokeratin; CD: cluster of differentiation; CEA: carcinoembryonic antigen; *PTTG-1*: *pituitary tumor-transforming gene 1*

Immunohistochemistry was performed. The colonic epithelium was reactive for cluster of differentiation (CD)20 and negative for cytokeratin (CK)7 (Figure 5a). CEA was positive in the epithelium (Figure 5b). Vimentin and actin were positive in the muscular layer and in the external and fibrous pseudocapsule (Figure 5c), CD56 (Figure 5d), and CD57 and CD99 were positive in the neuroepithelial focus. Epithelial membrane antigen (EMA), AFP, human placental lactogen (hPL), and beta-human chorionic gonadotropin (β-hCG) were negative. The inflammatory layer that mines a Peyer’s patch was positive for CD45, CD20, and CD3 (Figure 5e). *Pituitary tumor transforming gene-1* (*PTTG-1*) was also positive in small cells mixed in the inflammatory layer (Figure 5f). MCT was diagnosed in two cases and immature teratoma in one. Of note, octamer-binding transcription factor 4 (OCT4) was positive in all cases, as well as tumor suppressor protein 53 (P53). These results are described in Table 3.

**Table 3:** Immunohistochemistry results. CK: cytokeratin; OCT4: octamer-binding transcription factor 4; GFAP: glial fibrillary acidic protein; CD: cluster of differentiation; AFP: alfa-fetoprotein; CEA: carcinoembryonic antigen; β-hCG: beta-human chorionic gonadotropin; hPL: human placental lactogen; *PTTG-1*: *pituitary tumor-transforming gene-1*; Ki-67: marker of proliferation; P53: tumor suppressor protein 53

Primary antibodies	Case one	Case two	Case three
CK6/7/8	+	+	+
CK20	++	+	+
CKAE12/13	+	+	+
OCT4	++	+	+
Vimentin	+++	++	++
GFAP	Negative	Negative	+
CD56	Negative	Negative	+
CD99	Negative	Negative	++
AFP	+	+	+
CEA	+	+	+
B-hCG	+	Negative	Negative
hPL	+	+	+
CD3	+	+	+
CD20	+	+	+
CD45	++	++	++
PTTG-1	+	+	++
Ki-67	3	3	5
P53	++	++	++

## Discussion

Teratomas occur more frequently in children and young adults than in the older population and are more predominant in males compared to females. Teratoma is histologically formed by three germ cell layer tissues, namely, endoderm, ectoderm, and mesoderm (most teratomas are overweight). They are classified as mature or immature subtypes [1]. Teratoma is rare in hypophysis, with few published cases [2-5].

In this article, we reported three cases of sellar teratoma in a 65-year-old man with a history of chronic alcoholism, a 50-year-old man, and a 34-year-old young woman with menarche at nine years, irregular menstrual cycles, and four deliveries. During her last pregnancy, she developed visual disturbances that subsided after delivery. It is noteworthy that she could not breastfeed. All patients presented with a very similar clinical picture of progressive fatigue until drowsiness, visual disturbances, chiasmatic syndrome, and a large sellar lesion classified as Harvey IIIC and D, respectively. This type of tumor is more frequent in young women than in men. In all cases, the anterior pituitary hormone levels were normal. The female patient presented with a post-surgical middle cerebral artery infarction and passed away. In our cases, this tumor was more aggressive in women than in men, as the woman passed away quickly after surgery.

Teratoma diagnosis can be suggested through neuroimaging findings. MRI remains the preferred modality for assessment. As mentioned, neuroradiological findings are those of mixed cystic and solid components or inclusions of teeth, fat, and calcification. In our cases, cystic and solid structures with heterogeneous components not specific to fat, bone, or teeth were observed [5]. Nevertheless, the differential diagnosis including all primary and secondary sellar tumors related to pituitary adenomas may affect the sellar region and mimic pituitary tumors. These tumors are solid as well as cystic, and the final diagnosis is confirmed by biopsy and histological analysis [1]. Other sellar masses include cystic and solid lesions such as craniopharyngioma, tumor cyst, benign cyst, Rathke cyst, and other less common lesions such as aneurysm, squamous cell carcinoma, and metastases [1]. Cystic lesions within the sella turcica are not uncommon and may seem clinically and radiologically as a pituitary adenoma. Furthermore, metastasis to the pituitary gland is extremely rare; however, 1% of all pituitary tumors treated are derived from malignant neoplasms. Breast and lung cancers are the most common diseases that metastasize to the pituitary [6,7].

The ideal treatment for mature teratoma is neurosurgical excision [4,5]. However, there is a risk of damage to vessels directly involved with the tumor, commonly those of the internal carotid arteries, anterior cerebral arteries, as well as vessels outside of the surgical field of view, but adherent to the tumor capsule [6]. Failure to appreciate the tumor-vessel relationship may result in vasospasm and hemorrhage during or after transsphenoidal resection [5].

Pituitary apoplexy (PAp) is a severe and hypothetically life-threatening medical emergency [5], categorized by a constellation of symptoms or signs that occur as a result of acute hemorrhage and/or infarction in the pituitary gland [5]. Patients present with acute and sudden onset of symptoms or signs, most commonly with severe headache, vision deficits or ophthalmoplegia, altered mental status, and possible panhypopituitarism [6]. Patients should be monitored for complications related to endocrine function, as well as vasospasm and postoperative bleeding [5]. These advancements increase the likelihood of gross total resection and have resulted in decreased patient morbidity [6]. All our cases presented as medical emergencies and PAp was preliminarily diagnosed.

Tumor markers such as β-hCG and AFP can be useful for diagnosing teratoma as well as for distinguishing immature teratomas, mixed GCTs, and mature teratomas from immature or malignant components [1-4]. In our cases, the possibility of metastatic tumors was ruled out, and hormonal tumor markers were not analyzed, considering GCTs as the first option. Furthermore, tumoral markers were not assessed because a teratoma diagnosis was not made. However, clinical suspicion must always exist.

The mature subtype has all mature elements, containing adipose tissue, hair, sebaceous glands, and stratified squamous epithelium. The immature subtype contains immature epithelial, neuroepithelium, mesenchymal elements, or blastemal. Depending on the degree of differentiation of its components, immature tumors are more likely to exhibit malignant transformation [1,5]. We report a rare finding of mature intestinal tissue in all cases. Immunohistochemical staining can be used for differential diagnosis between MCT with colonic wall and mucinous adenocarcinoma or neurenteric cyst [8]. The microscopic and immunohistochemical patterns of the epithelium from the transitional zone between colonic wall-like structure and mucinous cystadenoma confirmed structures of both types of epithelium, positive for both CK7 and CK20 and focally positive for a neuroendocrine marker, chromogranin, which is normally present in colonic mucosa [8]. These results suggest that the mucinous cystadenoma originated from the colonic epithelium of the mature cystic [8].

Specific types of GCTs produce biological tumor markers such as AFP and/or hCG, and choriocarcinoma produces hCG. In teratomas, there are no specific markers, while they can express CK7 [7]. CKs also differentiate normal colons from carcinomas having positivity to CK20 and P53 and negativity for CK7 [8]. Similar to the benign mucinous epithelium, malignant epithelium associated with MCT more frequently shows positivity for CK20, MUC2, CDX2, ACE, CA19-9, and CK20 and is negative for CK7. Furthermore, ACE and cancer antigen 19-9 have been reported to have a strong relationship with malignant mucinous epithelium, or with malignant transformation [8].

Malignant changes or transformation and recurrence of mature intracranial teratoma are extremely rare. It is estimated that approximately 2% of all MCT cases show a malignant transformation. Squamous cell carcinoma is the most common type of tumor accounting for 83%, the rest are adenocarcinoma, sarcoma, and carcinoid tumors [9]. Adenocarcinoma arises from intracranial recurrent mature teratoma and features mutated *KRAS* and wild-type *BRAF *genes [10]. Conditions in traditional oncogenic pathways have been involved in the pathogenesis of sporadic pituitary adenomas. Constitutive expression of an isoform of the fibroblast growth factor receptor 4 has been associated with the pathogenesis of non-functioning pituitary adenomas [10]. Similarly, improved expression of *PTTG* has been detected in both functional and non-functional pituitary adenomas as well as in teratomas [11]. 

It is interesting to mention that cases of ovarian teratomas that present pituitary tissue and/or the combination of both teratomas with pituitary adenoma or normal pituitary tissue have been reported [12]. *PTTG-1* has been used to identify pituitary changes in teratomas [11], as well as in metastasis pituitary adenoma.

## Conclusions

We presented three rare cases of pituitary teratoma in patients of different ages and gender, but with the same symptoms. Only one case presented with endocrine disorders, associated with non-lactation and elevated prolactin. All patients had a rapidly evolving clinical picture and similar histological findings. Despite being benign, the prognosis for this tumor appears to be poor, as two patients deceased within a period of six months after therapy.
